# The Mediating Role of Resilience in the Relationship between Perceived Stress and Mental Health

**DOI:** 10.3390/ijerph18189762

**Published:** 2021-09-16

**Authors:** Mariela Loreto Lara-Cabrera, Moisés Betancort, C. Amparo Muñoz-Rubilar, Natalia Rodríguez Novo, Carlos De las Cuevas

**Affiliations:** 1 Department of Research and Development, Division of Mental Health, St Olav’s University Hospital, 7006 Trondheim, Norway; 2 Department of Mental Health, Faculty of Medicine and Health Sciences, Norwegian University of Science and Technology (NTNU), 7091 Trondheim, Norway; 3 Nidelv Division of Psychiatry, Community Mental Health Centre, St. Olav’s University Hospital, 7006 Trondheim, Norway; 4 Department of Clinical Psychology, Psychobiology and Methodology, Universidad de La Laguna, 38200 San Cristóbal de La Laguna, Spain; moibemo@ull.edu.es; 5 Faculty of Health Sciences, Nursing School, Universidad Central de Chile, Santiago 8370178, Chile; cristina.munoz@ucentral.cl; 6 Department of Nursing, Universidad de La Laguna, 38200 San Cristóbal de La Laguna, Spain; nrodrigu@ull.edu.es; 7 Colegio Oficial de Enfermeros de Santa Cruz de Tenerife, 38201 San Cristóbal de La Laguna, Spain; 8 Department of Internal Medicine, Dermatology and Psychiatry, Universidad de La Laguna, 38200 San Cristóbal de La Laguna, Spain; ccuevas@ull.edu.es; 9 Instituto Universitario de Neurociencia (IUNE), Universidad de La Laguna, 38200 San Cristóbal de La Laguna, Spain

**Keywords:** perceived stress, resilience, nurses, COVID-19 pandemic, descriptive survey study

## Abstract

The COVID-19 pandemic has created great uncertainty around the world, and due to the pandemic, nurses have been exposed to an increase in highly stressful clinical situations. This study examines the relationships between perceived stress and emotional disorders among nurses who have provided direct patient care during the COVID-19 pandemic and explores the mediating role of resilience in these relationships. In an online cross-sectional design, we asked Spanish nurses (*N* = 214) to complete self-reported scales, and we performed correlation and mediation analyses between perceived stress (Perceived Stress Scale, PSS-4), resilience (Wagnild Resilience Scale, RS-14), wellbeing (World Health Organization Wellbeing Index, five items, WHO-5), anxiety (PHQ-2) and depression (GAD-2). The nurses self-reported moderate levels of perceived stress, considerable psychological distress and high resilience. We found resilience to be significantly negatively correlated with the reported levels of perceived stress, anxiety and depression (*p* < 0.001). The mediating analysis revealed that resilience played a protective role in the direct relationships of stress with depression, anxiety, and psychological distress. To conclude, our findings supported the hypothesis that resilience mediated the relationship between stress and mental health.

## 1. Introduction

On 12 January 2020, the World Health Organization (WHO) confirmed that SARS-CoV-2 was the cause of a severe acute respiratory syndrome in a cluster of people in Wuhan, China. At the end of January 2020, the virus was first confirmed to have spread to Spain, and community spread in Spain was confirmed by mid-February. This led to an imposed lockdown that began on 14 March 2020 (state of alarm) [1]. During the week that followed the nationwide lockdown, the public health system became stressed and overwhelmed as never before [2].

Nurses constitute the largest workforce within healthcare systems globally and are pivotal to any coordinated response to public health emergencies, such as the COVID-19 pandemic [3]. The COVID-19 pandemic is a time of great uncertainty, and nurses have been exposed to an increase in highly stressful clinical situations. In Spain, nurses have been stretched to the point of exhaustion. They have been faced with supply shortages of personal protective equipment (PPE), which has led to the reuse of PPE despite the known health risks of this practice [4]. Due to this lack of PPE, nurses are often confined to quarantine after having been exposed to infected patients, and this has left many hospitals short-staffed. After years of austerity with resultant low salaries [2], the current situation has left nurses overworked with little financial reward. Furthermore, nurses may experience a variety of psychological problems [5,6,7], insomnia and stress [8], as well as the fear of being infected or transmitting the disease to their families when they provide care to COVID-19 patients [9]. Recent scientific literature has generated evidence that highlights the impact of the occupational stress that nurses have been facing during the pandemic on their mental health [8,10,11]. While some nurses who face COVID-19 pandemic stressors seem to cope effectively, others struggle. One relevant factor that can explain why some nurses possess the capacity to cope effectively with stress and the ability to bounce back from stressors is resilience [12].

Resilience refers to a person’s ability to withstand or adaptively recover from stressors [13]. Nurse resilience is defined as a complex and dynamic process which enables nurses to positively adapt to workplace stressors, avoid psychological harm and continue to provide high-quality patient care [14]. Protective factors play an important role in an individual’s risk of mental disorders, and resilience is one of the key protective factors in mental health [15]. Moreover, resilience protects against negative psychological outcomes [12,15], and has been found to be an essential protective factor for adaptive responses during stressful situations, such as a pandemic [16]. Resilience has been studied as a mediator. For instance, it has been suggested that resilience partially mediated the relationships between mental health and pandemic fatigue [17], and between emotional labour and depressive symptoms [18], as well as promoting psychological wellbeing [19]. As such, it is plausible to assume that resilience can mediate the relationship between stress and mental health problems among nurses. Therefore, the present study attempts to extend the previous literature by investigating two primary objectives. Firstly, we assessed the levels of perceived stress, resilience, wellbeing, and mental health in nurses providing direct patient care during the COVID-19 pandemic and investigated any possible associations between these variables. Secondly, we examined the potential mediating role of resilience on the nurses’ levels of perceived stress, depression, anxiety and psychological distress. Our research process is structured around the following hypotheses: nurses’ perceived stress is correlated with symptoms of anxiety and depression; resilience is negatively correlated with self-reported anxiety, depression and perceived stress; and finally, resilience acts as a mediator between perceived stress, symptoms of anxiety, depression and psychological distress.

## 2. Materials and Methods

We used an online survey to collect the data that we analysed in this cross-sectional study. We obtained the data during the Spanish COVID-19 state of alarm (June 2020) from nurses who were actively providing direct patient care on Tenerife, in the Canary Islands.

### 2.1. Participants, Procedures and Data Collection

We recruited nurses to participate in the online survey by sending a welcoming email containing a hyperlink to the survey to all the nurses enrolled in the Official College of Nursing of Tenerife. The purpose of this organisation was to regulate the Spanish nursing profession; it represented all of the registered nurses in Tenerife, and it ensured that proper standards were upheld and promoted ethical nursing practices. Two hundred and fourteen nurses met the inclusion requirements for working in the Canary Island of Tenerife and providing direct patient care during COVID-19 quarantine. The Research Ethics Committee of the Canary Islands Health Service approved all study procedures and materials (code CHUC_2020_33) and the participants provided informed consent prior to participation.

### 2.2. Demographics and Measures

We asked the participants to anonymously provide their socio-demographic information through self-reporting as part of the different questionnaires in the online survey. All the items were set as voluntary.

We used the Spanish version of the Wagnild Resilience Scale of 14 items (RS-14) to measure individual resilience [20]. The 14 items in this scale were scored on a 7-point Likert-type response format that was graded from ‘1’ (strongly disagree) to ‘7’ (strongly agree). The RS-14 measured 5 main characteristics of resilience: self-reliance, meaningfulness, equanimity, perseverance, and existential aloneness, and the item scores were added up to provide a total score ranging between 14 and 98, with higher scores suggesting a greater perceived resilience. According to a previous review study, scores below 65 indicated low resilience; those between 65 and 81 showed a moderate resilience; and scores higher than 81 were interpreted as high levels of resilience [15].

We employed the World Health Organization’s Wellbeing Index (five items; WHO-5) to measure participants’ degrees of positive wellbeing over the previous 2 weeks [21,22]. The scale’s five items were scored on a 6-point Likert scale (0 = at no time; 5 = all of the time). The total scores ranged between 0 and 25. This score could be transformed to an index score between 0 to 100 with higher scores indicating an increased sense of wellbeing. According to a clinimetric review of the WHO-5, a score below 13 indicated poor wellbeing and was an indication to test for depression [22]. The WHO-5 showed good internal consistency and construct validity in a Spanish sample [21]. 

The Perceived Stress Scale of 4 items (PSS-4) was used to assess psychological stress [23,24]. The PSS-4 was a brief self-report scale made up of four items that assessed the extent to which subjects perceived that their life over the past month had been unpredictable, uncontrollable, and overloaded. The response format consisted of a 5-point Likert scale (0 = never, 4 = very often). A higher score indicated a higher presence of perceived stress [24]. A mean of 5.4 constituted a valid reference for perceived stress in Spain [23]. 

The Patient Health Questionnaire of 2 items (PHQ-2) was a questionnaire that we used to measure depressive symptoms over the past 2 weeks [25]. PHQ-2 generated screening scores for depression. The scores for this questionnaire ranged between 0 and 6, and a score of 3 or greater was considered clinically relevant for depression [26].

The 2-item Generalized Anxiety Disorder screening tool (GAD-2) was used to measure anxiety [27]. The GAD-2 was a shorter version of the GAD-7 that used only the first two questions, which represented the core anxiety symptoms. The sum scores ranged between 0 and 6, and a score of 3 or greater was considered positive for anxiety screening purposes [26].

GAD-2 and PHQ-2 scores could also be calculated to determine a score for psychological distress by summing the two anxiety items and the two depression items, with scores ranging between 0 and 12 [28]. Higher scores indicated greater psychological distress, with the categories of psychological distress ranging from none (0–2), to mild (3–5), moderate (6–8), and severe (9–12) [28,29].

### 2.3. Statistical Analyses

We described participants’ characteristics at baseline by frequencies (%) or means (SD), depending on the distribution of each variable, and we used the Pearson correlation coefficient to evaluate the association between the scores. The statistical significance level was set at 0.05. The statistical packages used for the present study were SPSS version 25 (IBM Corp., NY, USA) and Lavaan package in R [30]. Mediation analyses were conducted with R [30], guided by a priori hypothesis testing [31].

Our independent variable was stress, while wellbeing, anxiety and depression were the dependent variables and resilience was the expected mediating or moderating variable. We estimated three mediation models using self-reported stress as the independent variable, psychological distress, anxiety and depression as the dependent variables and resilience as the mediation variable. Following recommended procedures, we calculated the maximum likelihood estimator and standard error using 1000 boot-strapped samples. To determine whether the indirect effects could be considered statistically significant, we inspected the *p*-values and ensured that the 95% confidence interval did not include zero [32].

## 3. Results

A total of 376 informants opened the survey, and 332 of these informants returned the questionnaires. The analysis sample included 214 nurses. Table 1 shows the sample’s sociodemographic characteristics and the results of the self-reported measures. Our sample of nurses consisted of 37 males (17.3%), 164 females (76.6%), and 13 participants (6.1%) who preferred not to report their gender. The mean age of the participants who chose to report their age (201/214, 87.6%) was 40.3 0 ± 11.6 years (ranging between 27 and 62 years). Twenty-five of the participants (11.7%) lived alone, and 42 (19.6%) had children in their care. Thirty-two of the participants (15.0%) lived with people with chronic diseases, while 28 (13.1%) lived with someone who was elderly. The mean wellbeing score (transformed) was 49.6.

More than three quarters of the nurses reported that most of the time they felt confident in their abilities to handle the COVID-19 crisis, while almost half self-reported that that, most of the time, their concern of acquiring the coronavirus had increased their stress level. Furthermore, more than half of the participating nurses self-reported moderate or high levels of perceived stress; 43.5% self-reported a high level of resilience; more than half of the participating nurses self-reported poor wellbeing; and finally, our findings showed that almost a third (31%) of the nurses experienced moderate or severe psychological distress.

### 3.1. Correlation Analysis

Table 2 presents an overview of the descriptive statistics and the correlations between the perceived stress (PSS-4), resilience (RS-14), wellbeing (WHO-5), anxiety (GAD-2), depression (PHQ-2) and psychological distress (PHQ-4) variables. Information about *p*-values is giving by using asterisks to mark significant levels (e.g., *p* < 0.001). Cronbach’s alphas appear in the diagonal between parentheses. According to the Pearson correlations we registered, the participants’ self-reported levels of stress correlated negatively with resilience and wellbeing and positively with psychological distress, anxiety and depression. 

Self-reported stress correlated significantly with psychological distress (r = 0.71, *p* < 0.001), anxiety (r = 0.62, *p* < 0.001) and depression (r = 0.65, *p* < 0.001). The nurses who perceived a high level of stress also indicated more psychological distress and more symptoms of anxiety and depression. These correlations confirmed our hypothesis that stress levels were positively correlated with psychological distress, anxiety and depression.

Resilience correlated positively with wellbeing and negatively with psychological distress, anxiety and depression. The resilience to wellbeing Pearson correlation of 0.49 indicates a moderate-to-high correlation between both variables, with nurses who scored high in resilience also scoring high in wellbeing. The Pearson correlations of resilience to anxiety (−0.499), depression (−0.564) and psychological distress (−0.590) indicated high correlations between resilience and these psychopathological indexes, and the nurses who scored low in resilience scored high in these three psychopathological indexes. Thus, the hypothesis that resilience was negatively correlated with psychological distress, symptoms of depression and anxiety could also be accepted. The hypothesis that there was a negative correlation between stress and resilience was also confirmed, with the correlation that was revealed between stress perceived and resilience (r = −0.62, *p* < 0.001), indicating that nurses who self-reported high levels of stress also reported low resilience.

### 3.2. Mediation Analysis

A series of partial mediation analyses were carried out to study the effect of resilience as a mediating variable in the relationship between stress as a predictor variable and depression, anxiety and mental distress as criterion variables. We estimated three mediation models using self-reported stress as the independent variable, psychological distress, anxiety and depression as the dependent variables and resilience as the mediation variable (as justified in the introduction).

Using resilience as the dependent variable and stress as the independent variable, the mediation model revealed that resilience decreased as stress increased (β = −2.39, std Error = 0.209, t = −11.47, *p* < 0.001, R^2^ adj. 0.38). The model that examined depression as the dependent variable (see Table 3) revealed a direct path from stress to depression (β = 0.24, *p* < 0.001, 95% CI = [0.18, 0.31]). We also found an indirect significant effect of resilience for this model (β = 0.08, *p* < 0.001, 95% CI = [0.04, 0.12]), which meant that resilience mediated this direct link. We found a reliable direct path between stress and anxiety (β = 0.26, *p* < 0.001, 95% CI = [0.20, 0.33]) in the model that tested the mediation of stress on anxiety. Resilience also mediated this link (β = 0.06, *p* < 0.001, 95% CI = [0.16, 0.1]). Finally, we found that the mediation role of resilience in the direct impact of stress on psychological distress showed a direct path from stress to personal distress (β = 0.50, *p* < 0.001, 95% CI = [0.40, 0.61]). Resilience also mediated this direct link, as we found an indirect significant effect of resilience (β = 0.14, *p* < 0.001, 95% CI = [0.07, 0.22]) (see Table 3 and Table 4).

If we take the three models together, we find that resilience seems to play a protective role in the direct relationships of stress with depression, anxiety and psychological distress. According to the estimated parameters (i.e., the betas in multiple regression mediation models) and the effect sizes, it seems that the protective role of resilience is higher for psychological distress compared to the average causal mediation effect value (ACME) for the three indirect effects. 

## 4. Discussion

Recent meta-analysis studies have revealed the strong impact that the COVID-19 outbreak has had on people’s psychological wellbeing and mental health [11,33], which increases the urgency of addressing mental health during and after this global health crisis. Through their daily routines, as well as during the exceptional circumstances brought about by the COVID-19 pandemic, nurses have a frontline position that is critical to the function of healthcare systems, as they are responsible for providing comprehensive care to all types of patients. Nurses have endured this health crisis in the face of an overload of work, generally precarious safety conditions due to lack of equipment and resources, and heightened anxiety regarding the risk of contagion for themselves and their loved ones, while their professional dedication to patient care holds them to standards that undermine their own strength [3]. 

With regard to our first research question concerning the levels of stress and symptoms of anxiety and depression nurses experienced, our findings showed that nurses who provided direct patient care during the COVID-19 pandemic in the Canary Islands, Spain, reported a considerable prevalence of perceived stress and symptoms of anxiety and depression. These results were in line with recent studies that revealed that the prevalence of anxiety and depression disorders was relatively high among healthcare workers during the COVID-19 pandemic [11,33,34,35]. Our results confirmed our first hypothesis, which posited that nurses’ perceived stress was positively correlated with symptoms of anxiety and depression. Similar to other studies, our analyses found that self-reported perceived stress correlated significantly with psychopathology [36,37], suggesting that nurses who perceived a high level of stress also perceived more symptoms of anxiety and depression. Remarkably, the levels of wellbeing among nurses during COVID-19, were even lower than among patients in the Spanish community mental health setting [21], and were lower than previous studies among nurses before and during COVID-19 [38,39]. 

The second hypothesis we tested was also confirmed, as we found that resilience was negatively correlated with self-reported anxiety, depression, and perceived stress. Our results showed that nurses with lower resilience are at higher risk of experiencing stress and symptoms of anxiety and depression. These findings are similar to those from studies that looked at nurses working in a COVID-19 unit in South Korea [40] and UK nurses working in a respiratory environment [41], as well as studies conducted in work settings before the COVID-19 pandemic. In terms of the non-COVID studies, there is evidence that resilience mediates psychological distress [42]. As such, this study extended previous knowledge of the importance of resilience among nurses. The literature shows, furthermore, that nurses in the present study reported moderate levels of resilience. This finding is consistent with previous studies among other populations, e.g., Brazilian medical students [43] and the general Spanish university population [20]. As resilience is one of the key protective factors in mental health [15], nurses reporting low levels of resilience could benefit from receiving effective psychosocial interventions that lead to long lasting improvements in resilience [12]. These types of interventions may be also useful for preventing the recurrence of psychopathology [11].

Finally, our third hypothesis regarding the role of resilience as a mediator between perceived stress and symptoms of anxiety, depression and psychological distress was also confirmed through the mediating analysis. This analysis confirmed that resilience seemed to play a protective role in the direct relationships of stress with depression, anxiety, and psychological distress. The negative effects of perceived stress on nurses’ psychopathology could be buffered directly and indirectly by resilience. These findings were consistent with earlier research [44] and contributed to a better understanding of nurses’ mental health in the face of the COVID-19 pandemic. They also provided references and guidance for the development of psychological support and mental health interventions to increase the resilience of nurses in times of crisis.

Our study has some limitations related to the methodology that should be taken into consideration. Firstly, our results may be affected by a selection bias, as there may be differences between the nurses who agreed to participate and those who did not. Secondly, the self-reported questionnaires we used to assess perceived stress, resilience and psychological distress are subject to the risk of response bias. Third, cross-sectional and mediational studies do not test causality directly, though they do provide evidence that may guide stakeholders and nursing administrators in supporting nurses. Fourth, even though our findings are limited to the study’s geographical context, they provide directions for future research aiming to examine resilience and mental health among nurses working during a pandemic.

## 5. Conclusions

The present study provides empirical evidence and considerable insight into the role and advantages of resilience in a sample of clinical nurses facing the COVID-19 pandemic. We found that resilience had a meaningful mediating effect that seemed to be determinant in perceived stress and symptoms of anxiety, depression and decreasing psychopathological symptoms in our sample. The knowledge generated by this study could be used to develop strategies for strengthening nurses’ resilience to prepare them for facing future stressful situations, as well as setbacks in their professional life. In these hard times, healthcare organisations must support nurses by avoiding exhaustive workloads and a lack of human and material resources, and maximise support for nurses who are experiencing high levels of stress to promote their wellbeing.

## Figures and Tables

**Table 1:** Characteristics of Study Participants.

Characteristics	Global (*N* = 214)
Female, *n* (%)	164 (76.6)
Mean age (SD ^1^)	40.3 (11.6)
Living arrangement, *n* (%)	
Alone	25 (11.7)
With other adults	141 (65.9)
With their children	42 (19.6)
With people with chronic diseases	32 (15.0)
With the elderly	28 (13.1)
Beliefs about coronavirus COVID-19, *n* (%) I feel confident in my abilities to handle this COVID-19 crisis	
Never	3 (1.4)
Some days	40 (18.7)
More than half the days	83 (38.8)
Almost every day	80 (37.4)
Missing	8 (3.7)
The concern of acquiring the coronavirus has increased my stress level	
Never	24 (11.2)
Some days	92 (43.0)
More than half the days	32 (15.0)
Almost every day	58 (27.1)
Missing	8 (3.7)
Perceived stress (PSS-4), total score, mean (SD) Low (0–5) Moderate (6–10) High (11–16)	5.8 (3.2) 48.6% 43.9% 7.5%
Resilience (RS-14), mean (SD) Low (14–64) Moderate (65–81) High (82–98)	77.7 (12.6) 15.9% 40.7% 43.5%
Wellbeing (WHO-5), total score, mean (SD) Poor wellbeing (0–12) Adequate wellbeing (13–25)	12.4 (4.9) 50.5% 49.5%
Anxiety (GAD-2), mean (SD) Depression (PHQ-2), mean (SD)	2.5 (1.6) 2.1 (1.6)
Psychologcal distress (PHQ-4), mean (SD)	4.6 (3.0)
None (0–2)	25.2%
Mild (3–5)	43.9%
Moderate (6–8)	19.2%
Severe (9–12)	11.7%

^1^ SD: Standard Deviation. PSS-4 = Perceived Stress Scale 4; RS-14 = 14-Item Wagnild Resilience Scale; WHO-5 = World Health Organization Wellbeing Index; PHQ-2 = Patient Health Questionnaire-2 items; GAD-2 = Generalized Anxiety Disorder 2-items; PHQ-4 = Patient Health Questionnaire-4 items.

**Table 2:** Descriptive statistics and correlations between perceived stress, resilience, wellbeing, anxiety and depression.

Variables	M	SD	Min	Max	1	2	3	4	5	6
1. Stress (PSS-4)	5.8	3.2	0	15	(0.70)					
2. Resilience (RS-14)	77.7	12.6	42	98	−0.62 ^***^	(0.88)				
3. Wellbeing (raw score WHO-5)	12.4	4.9	0	22	−0.60 ^**^	0.494 ^***^	(0.88)			
4. Anxiety (GAD-2)	2.5	1.6	0	6	0.62 ^**^	−0.499 ^**^	−0.670 ^***^	(0.79)		
5. Depression (PHQ-2)	2.1	1.6	0	6	0.65 ^**^	−0.564 ^**^	−0.708 ^**^	0.621 ^***^	(0.83)	
6. Psychological distress (PHQ-4)	4.6	2.9	0	12	0.71 ^**^	−0.59 ^**^	−0.76 ^**^	0.90 ^**^	0.90 ^***^	(0.84)

** Significant correlation, *p* value < 0.05. *** Significant correlation, *p* value < 0.001. M: Mean, SD: Standard Deviation, PSS-4 = Perceived Stress Scale 4; RS-14 = 14-Item Wagnild Resilience Scale; WHO-5 = World Health Organization Wellbeing Index; PHQ-2 = Patient Health Questionnaire-2 items; GAD-2 = Generalized Anxiety Disorder 2-items; PHQ-4 = Patient Health Questionnaire-4 items.

**Table 3:** Parameters estimated for model 1 depression as dependent variable, model II anxiety as dependent variable and model III psychological distress as dependent variable.

Variables	Estimate	Std. Error	*t-*Value	*p* (>|t|)	R^2^ adj.
Model I					
Intercept	3.2788	0.7864	4.1692	0.001	0.46
Resilience (RS-14)	−0.0342	0.0084	−4.0799	0.001	
PST Total	0.2474	0.0325	7.6226	0.001	
Model II					
Intercept	2.915	0.825	3.535	0.001	
Resilience (RS-14)	−0.024	0.009	−2.722	0.001	0.403
PST Total	0.257	0.034	7.555	0.001	
Model III					
Intercept	6.1938	1.3151	4.7098	0.001	0.53
Resilience (RS-14)	−0.0581	0.0140	−4.1467	0.001	
PST Total	0.5046	0.0543	9.2959	0.001	

RS-14 = 14-Item Wagnild Resilience Scale.

**Table 4:** Direct, indirect and total effect for mediation models. Model 1 depression as dependent variable, model II anxiety as dependent variable and model III psychological distress as dependent variable. Bootstrap CI estimation.

Variables	Estimate	95% CI Lower	95% CI Upper	*p*-Value
Model I				
ACME	0.0816	0.0421	0.12	0.001
ADE	0.2496	0.1847	0.31	0.001
Total Effect	0.3312	0.2792	0.38	0.001
Prop. Mediated	0.2437	0.1227	0.39	0.001
Model II				
ACME	0.0567	0.0162	0.1	0.001
ADE	0.259	0.1964	0.33	0.001
Total Effect	0.3157	0.2657	0.37	0.001
Prop. Mediated	0.1793	0.0499	0.32	0.001
Model III				
ACME	0.14030	0.07240	0.22	0.001
ADE	0.50370	0.39640	0.61	0.001
Total Effect	0.64400	0.55530	0.73	0.001
Prop. Mediated	0.21960	0.11100	0.34	0.001

CI = Confidence Interval.

## Data Availability

The data presented in this study are available from the corresponding author upon reasonable request.
